# Bilateral Congenital Absence of the Radius

**Published:** 1915-10

**Authors:** 


**Bilateral Congenital Absence of the Radius.** E. R. Secord.

(Annals of Surgery, March, 1915, Arch. of Paed., July). The parents of this infant were apparently normal, but a careful examination of the mother showed thickening of the bones of the fingers. Three of the other 4 children were healthy, but 1 had a marked deformity of the arms and died in infancy. This one was covered with a typical papulo-squamous eruption of syphilis. Otherwise nothing was noted except the remarkable condition of the forearms. Both radii were absent, as proven by examination and by X-ray. The ulna on each side was present, the only deformity being the congenital absence of both radii. Secord considers the condition due to a congenital syphilis.

